# SARS-CoV-2 Infects Primary Neurons from Human ACE2 Expressing Mice and Upregulates Genes Involved in the Inflammatory and Necroptotic Pathways

**DOI:** 10.3390/pathogens11020257

**Published:** 2022-02-17

**Authors:** Hussin A. Rothan, Pratima Kumari, Shannon Stone, Janhavi P. Natekar, Komal Arora, Tabassum T. Auroni, Mukesh Kumar

**Affiliations:** Department of Biology, College of Arts and Sciences, Georgia State University, Atlanta, GA 30303, USA; hussin.rothan@pfizer.com (H.A.R.); pkumari1@gsu.edu (P.K.); sstone12@student.gsu.edu (S.S.); jnatekar1@student.gsu.edu (J.P.N.); karora@gsu.edu (K.A.); tauroni1@student.gsu.edu (T.T.A.)

**Keywords:** COVID-19, SARS-CoV-2, K18-hACE2 mice, neurons, neuropathogenesis, inflammation, necroptosis

## Abstract

Transgenic mice expressing human angiotensin-converting enzyme 2 under the cytokeratin 18 promoter (K18-hACE2) have been extensively used to investigate the pathogenesis and tissue tropism of severe acute respiratory syndrome coronavirus-2 (SARS-CoV-2) infection. Neuroinvasion and the replication of SARS-CoV-2 within the central nervous system (CNS) of K18-hACE2 mice is associated with increased mortality; although, the mechanisms by which this occurs remain unclear. In this study, we generated primary neuronal cultures from K18-hACE2 mice to investigate the effects of a SARS-CoV-2 infection. We also evaluated the immunological response to SARS-CoV-2 infection in the CNS of K18-hACE2 mice and mouse neuronal cultures. Our data show that neuronal cultures obtained from K18-hACE2 mice are permissive to SARS-CoV-2 infection and support productive virus replication. Furthermore, SARS-CoV-2 infection upregulated the expression of genes involved in innate immunity and inflammation, including IFN-α, ISG-15, CXCL10, CCL2, IL-6 and TNF-α, in the neurons and mouse brains. In addition, we found that SARS-CoV-2 infection of neurons and mouse brains activates the ZBP1/pMLKL-regulated necroptosis pathway. Together, our data provide insights into the neuropathogenesis of SARS-CoV-2 infection in K18-hACE2 mice.

## 1. Introduction

Coronavirus disease 2019 (COVID-19), caused by severe acute respiratory syndrome coronavirus-2 (SARS-CoV-2), continues to be a global concern. In addition, several variants of SARS-CoV-2 have been identified that may influence antibody treatment and vaccine efficiency [1,2,3,4]. Neurological complications, such as brain fog, loss of taste and smell, changed mental status and anosmia have been reported in some COVID-19 patients [5,6,7,8,9]. Studies have shown presence of meningitis, encephalitis, leukocytes infiltration and neuronal death in COVID-19 patients [6,10]. Evidence of SARS-CoV-2 neuroinvasion in COVID-19 patients’ brain autopsies has been demonstrated and the olfactory mucosa has been suggested as a route of entry [4,6,11,12,13]. Several studies have also reported that neurologic symptoms may result from the exacerbated systemic pro-inflammatory responses without a direct infection of the brain cells [9,13]. Angiotensin-converting enzyme 2 (ACE2), the entry receptor of SARS-CoV-2, has recently been demonstrated to be present in neurons and glial cells of different brain regions [5,14,15,16,17,18]. Studies using brain organoids derived from human pluripotent stem cell (hPSC) have shown the presence of the virus in neuronal cells [19,20,21,22,23]. In addition, anti-ACE2 antibodies can inhibit the SARS-CoV-2 infection of neuronal cells [19]. 

The K18-hACE2 mouse model is commonly used to study the pathogenesis of SARS-CoV-2 infection and to test the efficacy of anti-viral compounds and vaccines. These mice express human ACE2, the entry receptor of SARS-CoV-2 [24,25,26,27]. We previously reported that the infection of K18-hACE2 mice with SARS-CoV-2 results in a lethal disease associated with viral neuroinvasion and severe neuronal damage [28]. However, the molecular mechanism by which SARS-CoV-2 infection of neurons leads to acute encephalitis in K18-hACE2 mice remains unclear. The present study was undertaken to (i) investigate the permissiveness of neurons to SARS-CoV-2 infection, and (ii) evaluate the immunological response to SARS-CoV-2 infection in the CNS of K18-hACE2 animals and mouse neuronal cultures. Our data show that neuronal cultures obtained from K18-hACE2 mice are permissive to SARS-CoV-2 infection and support productive virus replication. In response to infection, genes involved in the innate immune response, inflammation and cell death were upregulated in the neurons. In addition, SARS-CoV-2 infection of mouse brains also resulted in increased expression of genes associated with the inflammatory and cell death pathways. 

## 2. Results

### 2.1. SARS-CoV-2 Infection of Primary Mouse Cortical Neurons 

**Figure 1:**
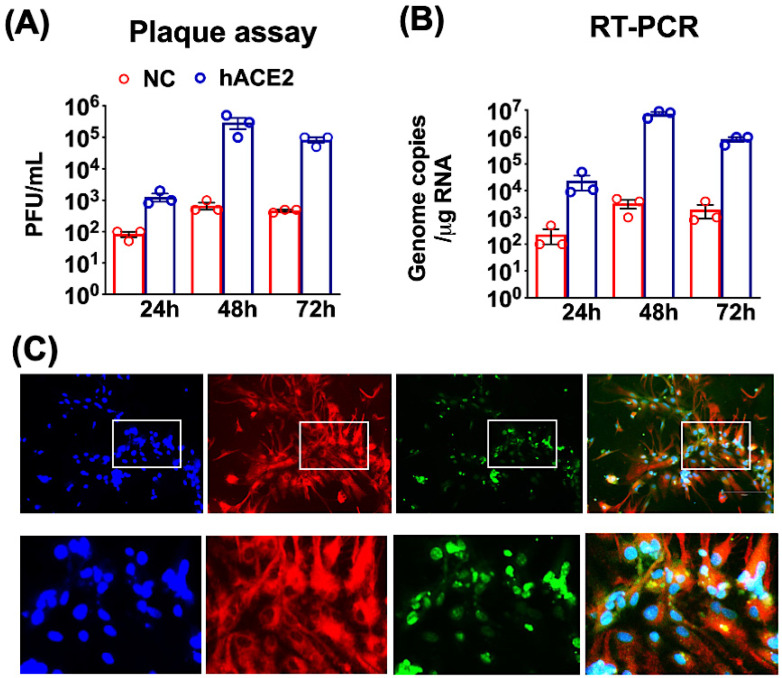
SARS-CoV-2 infection of mouse neuronal cultures. K18-hACE2 (hACE2 neurons) and non-hACE2-carrier (NC neurons) were prepared from one-day-old pups and cultured for seven days for differentiation. (**A**) hACE2 (blue bars) and NC neurons (red bars) were infected with SARS-CoV-2 at a MOI of 0.1. Virus infectivity titers in the supernatants were measured using a plaque formation assay and are expressed as plaque-forming units (PFU)/mL. (**B**) Intracellular viral RNA copies were determined by qRT-PCR. The data are expressed as genome copies/ug of RNA. Values are the mean ± SEM of three independent infection experiments conducted in duplicate. Each data point represents an independent experiment. (**C**) The hACE2 neurons grown on coverslips were fixed at 48 h after infection and stained with anti-MAP2 (red), dsRNA (green) and DAPI (blue) antibodies. In the bottom row of panels, the boxed areas from the first row are expanded. The images shown are representative of three independent infection experiments, with 20× magnification.

Primary neuronal cultures were established from one-day-old K18-hACE2 (hACE2 neurons) and non-hACE2-carrier (NC neurons) pups and cultured for seven days to allow differentiation to occur. The neuronal cultures were infected with SARS-CoV-2 at a multiplicity of infection (MOI) of 0.1. Plaque assay, qRT-PCR and immunofluorescence were used to determine the kinetics of SARS-CoV-2 replication at various time points after infection. Productive SARS-CoV-2 replication, as indicated by the release of virions, was detected at 24 h after infection of the hACE2 neurons. Viral titers peaked at 48 h after infection (log 5–6 PFU/mL), followed by a slight decrease in the virus titers at 72 h (Figure 1A). We next measured the intracellular viral RNA levels using qRT-PCR. High SARS-CoV-2 RNA levels were detected in the hACE2 neurons at 48 and 72 h after infection (log 6–7 genome copies/ug RNA). Neurons derived from NC mice were relatively resistant to infection compared to the hACE2 neurons. There was a slight increase in virus and RNA levels at 48 and 72 h, suggesting limited virus replication in these cells (Figure 1B). An immunofluorescence assay of SARS-CoV-2-infected hACE2 neurons showed strong dsRNA staining. Additionally, dsRNA was detected in both the neuronal bodies and axons of the MAP2-positive cells at 48 h after infection (Figure 1C). dsRNA detection is considered as evidence of viral RNA replication. Approximately 40% of hACE2 neurons were positive for dsRNA at 48 h after infection. Overall, these findings indicate that neurons derived from K18-hACE2 mice are permissive to SARS-CoV-2 infection and support productive virus replication. 

### 2.2. Host Immune Responses in SARS-CoV-2-Infected Neurons and Mouse Brains 

**Figure 2:**
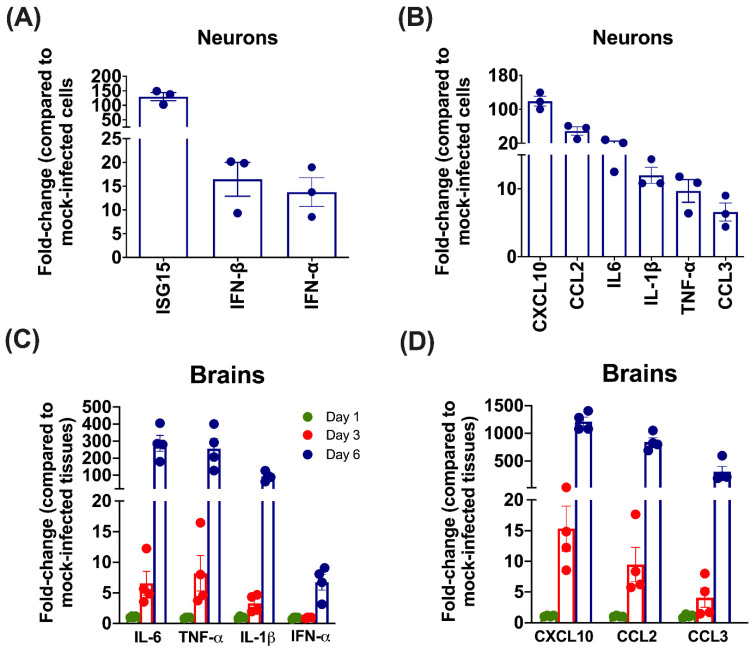
Analysis of upregulation of the expression of immune genes involved in innate immunity and inflammation in primary mouse neurons and mouse brains. (**A**,**B**) The hACE2 neurons were infected with SARS-CoV-2 or mock-infected at a MOI of 0.1. At 48 h after infection, cell pellets were collected, and total RNA was extracted. qRT-PCR was conducted to determine the fold-change of (**A**) ISG15, IFN-β and IFN-α, and (**B**) CXCL10, CCL2, IL-6, IL-1β, TNF-α and CCL3 mRNA levels. Data for each sample was normalized to the value for GAPDH and expressed as the relative fold increase compared to mock-infected controls. Data represent the mean ± SEM of three independent infection experiments conducted in duplicate. Each data point represents an independent experiment. (**C**,**D**) Eight-week-old hemizygous K18-hACE2 mice were infected with SARS-CoV-2 (10^4^ PFU, n = 12) or PBS (Mock, n = 9). Brains were harvested after extensive perfusion with PBS at days 1, 3 and 6 after infection and RNA was extracted. The mRNA levels of (**C**) IL-6, IL-1β, TNF-α and IFNα, and (**D**) CXCL10, CCL2 and CCL3 were determined by qRT-PCR. Each data point represents an individual mouse. Data represent the mean ± SEM.

We next investigated the effect of SARS-CoV-2 infection on the mRNA expression of key innate immune and inflammation genes in the neurons. Changes in gene expression levels in hACE2 neurons infected with SARS-CoV-2 for 48 h compared to mock-infected controls were analyzed by qRT-PCR. Interferon stimulated gene (ISG)-15 mRNA expression increased by >100-fold after SARS-CoV-2 infection (Figure 2A). The levels of interferon (IFN)-α and IFN-β were elevated more than 10-fold. The mRNA levels of the chemokine pathway-associated genes, chemokine (C-C motif) ligand-2 (CCL2) and chemokine (C-X-C motif) ligand-10 (CXCL10), were upregulated by more than 50-fold in infected neuronal cultures (Figure 2B). Furthermore, interleukin-6 (IL-6), IL-1β and tumor necrosis factor (TNF)-α mRNA expression levels were upregulated >10-fold by a SARS-CoV-2 infection. The CCL3 mRNA levels were also increased compared to the mock-infected controls (Figure 2B). 

Next, we examined the mRNA levels of innate immune and inflammatory genes in the brains of infected mice. K18-hACE2 mice infected with PBS or 10^4^ PFU of SARS-CoV-2 via the intranasal route [28]. The mice were sacrificed at days 1, 3 and 6 after infection, and the brains were harvested. A plaque assay was conducted to determine infectious virus titers in the brain homogenates. No infectious virus was detected in the brains on day one, but virus infectivity titers were very high at day 3 (log 3–4 PFU/gram of brain tissue) and day 6 after infection (log 7–8 PFU/gram of brain tissue) [28]. Both the IL-6 and TNF-α mRNA levels increased by >5-fold on day 3 after SARS-CoV-2 infection (Figure 2C). By the sixth day after infection, IL-6 and TNF-mRNA levels in the brain had increased by 300-fold (Figure 2C). There was also a 100-fold increase in the IL-1β mRNA levels on day 6. The was a slight upregulation in the levels of IFN-α mRNA (Figure 2C). At day 6 after infection, expression levels of chemokines, including CXCL10 and CCL2 and CCL-3 were elevated by more than 300-fold (Figure 2D). 

**Figure 3:**
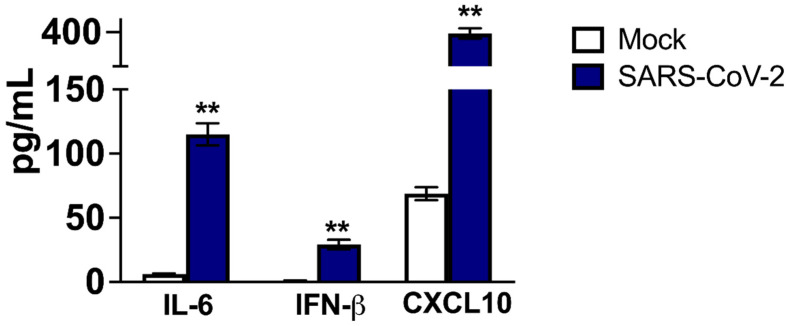
Protein levels of IL-6, IFN-β and CXCL10 in SARS-CoV-2-infected neurons: Supernatant collected from hACE2 neurons infected with SARS-CoV-2 or mock-infected for 48 h was used to determine the levels of IL-6 and IFN-β and CXCL10 using ELISA. The data expressed are the mean concentration (pg/mL) ± SEM of the amount of IL-6 and IFN-β and CXCL10 secreted in the supernatant and are representative of three independent experiments. ** *p* < 0.001.

As these pro-inflammatory cytokines are secreted proteins, their release in the culture media of mock- and SARS-CoV-2 -infected hACE2 neurons was detected using ELISA. In the controls, basal levels of IL-6 and IFN-β in cell culture media were very low. On the other hand, significant amounts of soluble IL-6 and IFN-β were detected in the supernatant from infected cells at 48 h after infection (Figure 3). The basal level of CXCL10 was relatively high, but it also increased significantly after SARS-CoV-2 infection. These results indicate that SARS-CoV-2 infection upregulates the expression of innate immune and inflammatory genes in neuron cultures and mouse brains. 

### 2.3. SARS-CoV-2 Infection Activates the ZBP1/MLKL Pathway in Neurons and Mouse Brains

**Figure 4:**
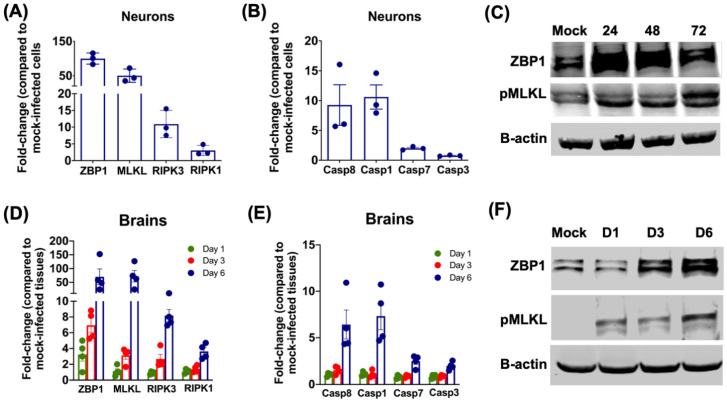
mRNA and protein levels of genes involved in cell-death pathways in primary mouse neurons and mouse brains. (**A**,**B**) The hACE2 neurons were infected with SARS-CoV-2 or mock-infected at a MOI of 0.1. At 48 h after infection, cell pellets were collected, and total RNA was extracted. qRT-PCR was conducted to determine the fold-change in (**A**) ZBP1, MLKL, RIPK3 and RIPK1, and (**B**) Caspase 8, Caspase 1, Caspase 7 and Caspase 8 mRNA levels. Data for each sample was normalized to the value for GAPDH and expressed as the relative fold increase compared to mock-infected controls. Data represent the mean ± SEM of three independent infection experiments conducted in duplicate. Each data point represents an independent experiment. (**C**) The hACE2 neurons were infected with SARS-CoV-2 or mock-infected at a MOI of 0.1. At 24, 48 and 72 h, cell pellets were collected, and total protein was extracted. Protein was blotted with ZBP1, pMLKL or β-actin antibodies. Data are representative of three independent experiments. (**D**,**E**) K18-hACE2 mice were inoculated with SARS-CoV-2 or PBS via the intranasal route. Brains were harvested after extensive perfusion at days 1, 3 and 6 after infection and RNA was extracted. qRT-PCR was used to determine the mRNA levels of (**D**) ZBP1, MLKL, RIPK3 and RIPK1, and (**E**) Caspase 8, Caspase 1, Caspase 7 and Caspase 8. After normalizing individual sample to GAPDH level, the fold change in infected tissues compared to mock-infected controls was determined. Each data point represents an individual mouse (n = 4). Values are the mean ± SEM. (**F**) Protein extracted from mock- and SARS-CoV-2-infected brain tissues were blotted with ZBP1, pMLKL or β-actin antibodies. Data are representative of four mice per time point.

We examined the mRNA and protein levels of genes involved in cell-death pathways in neurons after SARS-CoV-2 infection. The qRT-PCR was used to analyze the changes in the gene mRNA levels. The key genes involved in the necroptotic pathway were highly upregulated in hACE2 neurons infected with SARS-CoV-2 for 48 h. The levels of Z-DNA binding protein 1 (ZBP1) and mixed lineage kinase domain-like (MLKL) mRNA were elevated ~50-fold after SARS-CoV-2 infection. mRNA expression levels of caspase-8 and receptor-interacting kinase-3 (RIPK3) were upregulated >10-fold after infection (Figure 4A). Pyroptotic gene caspase-1 was upregulated by 10-fold while the apoptotic genes, caspase-3 and caspase-7 showed no significant increase after SARS-CoV-2 infection (Figure 4B). To verify the activation of the necroptotic pathway, protein levels of ZBP1 and phosphorylated MLKL (pMLKL) were measured by immunoblotting. The levels of ZBP1 increased at 24 and 48 h after infection. We detected a modest increase in the protein levels of pMLKL at 24 and 48 h after infection. However, there was a significant increase in the levels of pMLKL protein at 72 h (Figure 4C). 

Next, we evaluated the activation of the necroptotic pathway in mouse brains infected with SARS-CoV-2. The mRNA expressions of ZBP1 and MLKL increased gradually in the brains from days 1 to 6 after SARS-CoV-2 infection. By day 6 after infection, ZBP1 and MLKL mRNA levels were upregulated ~50-fold in the brains (Figure 4D). The mRNA levels of RIPK3, RIPK1, Caspase 8 and Caspase 1 were also elevated by day 6 (Figure 4D,E). However, there was no significant increase in the levels of caspase-3 and caspase-7 mRNA in infected brains. Western blot data showed an increase in the protein levels of ZBP1 and pMLKL in the infected brains in a time-dependent manner (Figure 4F). The increase in the mRNA and protein levels of ZBP1 and MLKL correlate with the increase in the infectious virus titers in the brains [28]. Together, these results indicate that SARS-CoV-2 infection in neurons and mouse brains activates the ZBP1/MLKL-regulated necroptosis pathway.

## 3. Discussion

In this study, we demonstrated that SARS-CoV-2 establishes a productive infection in neuronal cultures obtained from hACE2-expressing mice. In response to infection, the expression of innate immune and inflammatory genes was upregulated in the neurons as well as in mouse brains. In addition, we found that the SARS-CoV-2 infection of neurons and mouse brains upregulated genes involved in the necroptotic pathway (ZBP1, MLKL RIPK3 and caspase-8), suggesting that necroptosis may play a role in the pathogenesis of SARS-CoV-2 infection in the CNS.

It is known that some animal (mouse hepatitis virus) and human (HCoV-OC43) coronaviruses productively infect neuronal cells [28,29]. The SARS-CoV-2 infection has been detected in the brains of some COVID-19 patients [4,6,11,12,13]. Furthermore, SARS-CoV-2 has also been shown to replicate and induce cell death in human neural progenitor cells and brain organoids [19,20,21,22,23]. Transgenic K18-hACE2 mice represent a lethal model of SARS-CoV-2 infection [24,25,26,27,28]. Neuroinvasion and the replication of SARS-CoV-2 within the CNS is associated with mortality in these mice. In the present study, we found that neurons derived from one-day-old K18-hACE2 mice are permissive to SARS-CoV-2 infection and support productive virus replication. Additionally, dsRNA was detected in the neuronal bodies and axons infected with SARS-CoV-2. In comparison, virus replication was limited in the non-hACE2-expressing mouse neurons. 

A cytokine storm is one of the pathological hallmarks of the severe outcomes resulting from SARS-CoV-2 infection [30,31]. Several studies have reported that increased TNF-α and IL-6 levels correlate with severe disease outcomes [32,33]. In the present study, we show that SARS-CoV-2 infection in K18-hACE2 mouse brains is also characterized by the upregulation of innate immune and inflammatory genes, including TNF-α and IL-6 [28]. Similarly, a significant increase in the expression of IL-6, TNF-α, CXCL10 and CCL2 was observed in SARS-CoV-2-infected neuron cultures. These inflammatory genes may activate downstream cell-death signaling pathways in the neurons, leading to neuronal death, and/or stimulate glial cells, exacerbating neuroinflammation [33,34,35]. As previously reported, TNF-α is thought to be a potent inducer of neuronal injury in several neurodegenerative diseases, such as cerebral ischemia, spinal cord injury, multiple sclerosis and viral infections including HIV-associated dementia [33,36]. Both CXCL10 and CCL2 are important chemokines involved in the infiltration of leukocytes into the CNS after virus infection [35]. 

ZBP1 is one of the cytoplasmic sensors that regulate cell death and inflammation [37,38,39,40] and initiates the RHIM-dependent activation of RIPK3-dependent necroptosis during virus infections. Necroptosis is an inflammatory cell death caused by RIPK3 phosphorylation, which activates the pseudo-kinase MLKL, which oligomerizes and ruptures the plasma membrane, resulting in cell death [41,42]. Necroptosis can eradicate virus-infected cells and activate innate and adaptive immunity to limit virus replication. This process can also trigger the release of inflammatory cytokines and damage-associated molecular patterns, resulting in robust inflammation [38,39,43]. In the present study, we found significant upregulation of the necroptotic genes, ZBP1, MLKL and RIPK3, in neuronal cells and mouse brains after SARS-CoV-2 infection. Previous studies have demonstrated that infection with beta coronaviruses can induce necroptosis in certain cell types. Human coronavirus, HCoV-OC43, induces necroptosis in human neural cells [29] and mouse hepatitis virus infection induces necroptosis in murine bone-marrow-derived macrophages by phosphorylation of MLKL [44]. We previously reported that ZBP1 restricts replication of West Nile virus and Zika virus in primary mouse cortical neurons [37]. Future studies are warranted to understand the role of ZBP1 in SARS-CoV-2 pathogenesis. 

Together, our results demonstrate that SARS-CoV-2 robustly replicates in neuronal cultures obtained from K18-hACE2 mice. In the same way as the SARS-CoV-2-infected K18-hACE2 mouse brains, the virus infection of neuronal cultures induces the up-regulation of genes involved in the innate immune response, inflammation and cell death.

## 4. Materials and Methods

### 4.1. Neuronal Cultures and SARS-CoV-2 Infection 

Hemizygous K18-hACE2 mice and non-hACE2-carrier (NC) mice were purchased from the Jackson Laboratory (Bar Harbor, ME, USA). One-day-old pups were obtained from established breeding pairs of K18-hACE2 and NC mice as previously described [37,45,46]. The neurons were plated for 24 h onto poly-D-lysine-coated plates in serum Neurobasal A medium. The neurons were then cultured in serum-free Neurobasal A medium containing B27 for seven days to allow differentiation. The neurons isolated from each pup were plated separately and genotyped to identify hACE2-expressing and NC neurons. Neuronal cultures were infected with SARS-CoV-2 (USA-WA1/2020) or mock-infected at a multiplicity of infection of 0.1. At various time points after infection, supernatants and cell lysates were collected [37,45,47,48].

### 4.2. Animal Infection Experiments

The in vivo animal experiments with SARS-CoV-2 were conducted in an Animal Biosafety Level 3 (ABSL-3) laboratory. Georgia State University Institutional Animal Care and Use Committee approved the experimental protocol of this study (Protocol number A20044). Hemizygous K18-hACE2 mice aged eight-weeks were inoculated with PBS or 10^4^ PFU of SARS-CoV-2 via the intranasal route [28,48]. On various days after the infection, animals were anesthetized, perfused with PBS, and brain tissues were collected.

### 4.3. Quantification of the Viral Titers

The levels of infectious virus in cell culture supernatants and brain tissues were determined by using a plaque formation assay. Quantitative RT-PCR was used to measure the intracellular viral RNA levels using SARS-CoV-2 N gene primers [28,47]. Total RNA was extracted from cell pellets and brain tissues using a Qiagen RNeasy Mini kit (Qiagen, Germantown, MD, USA). Viral genome copies per ug of total RNA were calculated using a standard curve of the known amount of viral RNA [28,47,48].

### 4.4. Immunostaining 

Neuronal cells were grown on coverslips in 12-well plates and infected with SARS-CoV-2 or PBS at a MOI of 0.1 for 48 h [45]. Cells were washed with PBS and fixed in 4% paraformaldehyde for 1 h at room temperature. The cells were permeabilized and incubated with anti-MAP2 (Catalog # PA5-17646) and anti-dsRNA (MABE1134) antibodies overnight at 4 C (Thermo Fisher Scientific, Norcross, GA, USA). The next day, cells were incubated with Alexa Fluor 546- or Alexa Fluor 488-conjugated secondary antibody for 1 h at room temperature [28,45,49]. The Invitrogen EVOS™ M5000 Cell Imaging System was used to capture the images. 

### 4.5. Western Blot Analysis 

Protein extracted from neuronal cultures and mouse brains were separated on SDS-PAGE and transferred onto PVDF membranes. The membranes were incubated with primary antibodies against ZBP1 (Cat #703166), pMLKL (Thermo Fisher Scientific, Norcross, GA, USA) and β-actin [49,50]. To visualize the protein bands, the membranes were incubated with a secondary antibody conjugated with IRDye 800 or IRDye 680 (Li-Cor Biosciences). The membranes were scanned using the Odyssey infrared imager (Li-Cor Biosciences) [49,50].

### 4.6. ELISA 

ELISA was used to measure the protein levels of IL-6 (Invitrogen, Catalog # 50-246-676), IFN-β (PBL Assay Science, Catalog # 12405-1) and CXCL10 (Invitrogen, Catalog # 50-182-92) in the cell culture supernatants, according to the manufacturer’s instructions. The plates were analyzed using a Victor 3 microtiter reader as previously described [28].

### 4.7. qRT-PCR 

A Qiagen RNeasy Mini kit (Qiagen, Germantown, MD, USA) was used to extract total RNA from cell pellets and brains. The cDNA was synthesized from RNA using an iScript™ cDNA Synthesis Kit (Bio-Rad). The qRT-PCR was used to determine the expression levels of multiple host genes [51]. The fold-change in infected samples compared to control samples was calculated after normalizing to the housekeeping GAPDH gene [28,45,47,48]. The primer sequences used for qRT-PCR are listed in Table 1.

### 4.8. Statistical Analysis

Unpaired student *t*-test using GraphPad Prism 8.0 (GraphPad Software, San Diego, CA, USA) was used to calculate the *p* values. Differences of *p* < 0.05 were considered significant.

## Figures and Tables

**Table 1:** Primer sequences used for qRT-PCR.

Gene (Accession No.)	Primer Sequence (5′–3′)
IL-1β (NM_000576)	
Forward	AGCACCTTCTTTCCCTTCATC
Reverse	GGACCAGACATCACCAAGC
IL-6 (NM_000600)	
Forward	CCAGGAGCCCAGCTATGAAC
Reverse	CCCAGGGAGAAGGCAACTG
CCL3 (NM_011337)	
Forward	ATTCCACGCCAATTCATC
Reverse	ATTCAGTTCCAGGTCAGT
IFN-α (NM_010502)	
Forward	CTCTGTGCTTTCCTGATG
Reverse	CTGAGGTTATGAGTCTGAG
TNF-α (NM_013693)	
Forward	CCAGTCTGTATCCTTCTAA
Reverse	TCTTGTGTTTCTGAGTAGT
CCL2 (NM_011333)	
Forward	TCACCTGCTGCTACTCATTCACCA
Reverse	TACAGCTTCTTTGGGACACCTGCT
ISG-15 (NM_015783)	
Forward	AGAGCCACTGTTGGTTAT
Reverse	TTTCCTCGTTTACATTTCCA
Caspase 1 (NM_009807)	
Forward	GGAAGCAATTTATCAACTCAGTG
Reverse	GCCTTGTCCATAGCAGTAATG
Caspase 3 (NM_009810)	
Forward	ATCCTGAAATGGGCATAT
Reverse	CTTCCTTAGAAACACTATCC
Caspase 7 (NM_007611)	
Forward	GTGACACCCATAAAGGAT
Reverse	ATGCCTGAATGAAGAAGA
Caspase 8 (NM_009812)	
Forward	CTAGTTCTCTCAGTTGTCTTT
Reverse	GAGGTTTGCTACCGATTC
ZBP1 (NM_021394)	
Forward	GAAATAAGCACCTTCTGAG
Reverse	GAATTGGCAATGGAGATC
MLKL (NM_029005)	
Forward	GGAACTTAGGCTATGGATA
Reverse	CGGCAGTATTTCATCTTT

## Data Availability

Not applicable.
